# Endothelial Progenitor Cells: An Appraisal of Relevant Data from Bench to Bedside

**DOI:** 10.3390/ijms222312874

**Published:** 2021-11-28

**Authors:** Doralisa Morrone, Maria Elena Lucia Picoi, Francesca Felice, Andrea De Martino, Cristian Scatena, Paolo Spontoni, Antonio Giuseppe Naccarato, Rossella Di Stefano, Uberto Bortolotti, Massimo Dal Monte, Stefano Pini, Marianna Abelli, Alberto Balbarini

**Affiliations:** 1 Department of Surgical, Medical and Molecular Pathology and Critical Care Medicine—Cardiovascular Disease Section, University of Pisa, 56100 Pisa, Italy; picoielena@tiscali.it (M.E.L.P.); paolo.spontoni@gmail.com (P.S.); rossella.distefano@unipi.it (R.D.S.); a.balbarini@med.unipi.it (A.B.); 2 Department of Surgical, Medical and Molecular Pathology and Critical Care Medicine—Surgery Section, University of Pisa, 56100 Pisa, Italy; and.demartino@libero.it (A.D.M.); uberto.bortolotti@med.unipi.it (U.B.); 3 Department of Translational Research and New Technologies in Medicine and Surgery, University of Pisa, 56126 Pisa, Italy; cristian.scatena@unipi.it (C.S.); giuseppe.naccarato@med.unipi.it (A.G.N.); 4 Department of Biology, University of Pisa, 56100 Pisa, Italy; massimo.dalmonte@unipi.it; 5 Department of Clinical and Experimental Medicine, University of Pisa, 56100 Pisa, Italy; stefano.pini@unipi.it (S.P.); m.abelli@libero.it (M.A.)

**Keywords:** EPC, ischemic heart disease, progenitor cells

## Abstract

The mobilization of endothelial progenitor cells (EPCs) into circulation from bone marrow is well known to be present in several clinical settings, including acute coronary syndrome, heart failure, diabetes and peripheral vascular disease. The aim of this review was to explore the current literature focusing on the great opportunity that EPCs can have in terms of regenerative medicine.

## 1. Introduction

Cardiovascular disease (CVD) is one of the main causes of death and morbidity in Europe, with a huge economic burden on society. Additionally, as life expectancy rises, increased prevalence and complexity of these conditions is anticipated. The aetiology and progression of CVD is multifactorial, ranging from genetics to external factors, such as lifestyle, diabetes, obesity, hypertension, and hypercholesterolemia. Furthermore, there is increasing awareness of the relevance of depression and anxiety in the pathophysiology of CVD.

In recent decades, several pieces of evidence have supported the existence of an innovative pathway in which tissue residents or migrating progenitor cells contribute to cardiovascular (CV) self-repair through differentiation into CV cell lineages and paracrine/autocrine actions [1]. These endothelial progenitor cells (EPCs), isolated for the first time by Ashara and colleagues in 1997 [2], represent a heterogeneous population of hematopoietic and non-hematopoietic progenitor cells able to participate in neovascularization and vascular remodelling [3], with an important role in cardiovascular homeostasis and characterization of CVD.

## 2. The Physiology of Endothelial Progenitor Cells

Adult bone marrow contains at least two types of stem cells: hematopoietic stem cells (HSCs) and mesenchymal stem cells (MSCs) [4]. Asahara and collaborators firstly identified EPCs both in bone marrow and in peripheral blood where they can be isolated by magnetic bead selection on the basis of the CD34 antigen, and they were found to be positive for CD34, CD133, and vascular endothelial growth factor (VEGF) receptor 2 [2].

Since then, it has been widely accepted that EPCs originate in the bone marrow and can be mobilized into the peripheral blood either endogenously by tissue ischemia or exogenously by cytokine stimulation or HMG-CoA reductase inhibitors [5], thus differentiating into mature endothelial cells [6], contributing to the repair of vascular damage.

Prior to the discovery of EPCs, it was commonly believed that the formation of new vessels was totally dependent on the proliferation of resident endothelial cells (ECs). As described above, this dogma was rectified when Asahara and co-workers isolated CD34 + hematopoietic progenitor cells from adult peripheral blood capable of differentiating ex vivo into an endothelial phenotype. The authors succeeded in demonstrating that the vasculogenesis process could occur during embryogenesis and also in the adult individual.

Most EPCs reside in the bone marrow in a close relationship with hematopoietic stem cells (HSCs) and the medullary stroma, which provides an optimal microenvironment for hematopoiesis.

EPCs are a heterogeneous population characterised by the expression of surface markers of endothelial (vascular endothelial-cadherin or CD144; vascular endothelial growth factor receptor 2 or VEGFR-2 or KDR; CD31 and von Willebrand factor, vWF) and haemopoietic progenitor (CD34, CD133) lineages. EPCs derive from the bone marrow and can be mobilised into peripheral circulation in response to many stimuli, including tissue ischemia and vascular damage, through the release of growth factors and cytokines [7].

The ability of EPCs to promote the revascularization of ischemic and damaged tissues has been examined both in animal and in human models in the treatment of ischemia [8]. In the presence of endothelial dysfunction, EPCs are incorporated in the area of vascular damage, providing ECs for the growth of new vessels and stimulating the secretion of growth factors that activate adjacent cells. Several studies have shown that circulating EPC levels are modulated during ischemic damage as a consequence of various pathological states, by age and by the presence of risk factors for CVD such as smoking, hyperlipidaemia and diabetes [9,10]. In particular, it has been shown that the number and functional activities of EPCs are proportionally reduced with cardiovascular risk factors [11].

The term EPCs currently encompasses several different cell populations, each playing a different role in regeneration and neoangiogenesis. Controversies regarding their origin, ambiguity in the phenotyping and non-standardized isolation techniques have emerged alongside difficulties in the isolation of EPCs.

## 3. EPC Isolation and Characterization

The isolation and characterization of EPCs can be summarized into three basic techniques, which were later modified by various researchers.

### 3.1. In Vitro Culturing Techniques

Functional and biochemical analysis of EPCs isolated by culturing peripheral blood mononuclear cells (PBMCs) has established two sub-populations of cells with an endothelial phenotype: early and late EPCs, both of which have different morphologies, proliferation rates, survival behaviours, gene expression profiles and secreting activity leading to different functions in vitro [12]. PBMCs from healthy male donors were isolated by density gradient centrifugation according to the manufacturer’s protocol. 

The in vitro technique involves plating PBMCs on fibronectin-coated dishes for approximately four days. The non-adherent cells are then removed, leaving PBMCs on the dish. The four-day period is selected because the unwanted platelets, red blood cells, or monocytes are gradually depleted over this period. The number of days is, however, not fixed and has been modified by various researchers [13]. Early EPCs appear within four to seven days of culture, show a limited proliferating potential for long term culture and disappear after two weeks in in vitro conditions. They express both endothelial and monocytic markers (CD31+/CD144−/CD34−/CD14+/CD45+), have a low expression of endothelial nitric oxide synthase (eNOS) and VEGFR-2 [12,14] and release proangiogenic growth factors, as confirmed by transcriptomic data. Late EPCs develop from two to three weeks after plating and show a cobblestone appearance similar to mature endothelial cells, expressing only endothelial markers [12,15]. They show a long life span and rapidly replicate from several cells to a colony and become a monolayer. 

### 3.2. Molecular Techniques

Circulating EPCs are identified based on their expression of cell surface markers by the use of MACS (magnetic-activated cell sorting), which employs magnetic beads coated with the antibody/protein of choice [16], or by FACS (fluorescence-activated cell sorting), a specialized type of flow cytometry which works on the principle of excitation and emission of fluorochromes bonded to the antibody/protein. The CD133+/VEGFR-2+ cells are believed to be less mature or early circulating EPC, while more mature circulating EPC (late EPC), which have lost CD133, are positive for CD34, VEGFR-2, CD31, VE-cadherin, and eNOS [12]. Flow cytometry was utilized to investigate and enumerate the EPC concentration in the blood of patients at risk for or with clinical diagnosis of vascular disease [17]. However, the phenotypic classification of these cells is still controversial. In fact, some CD34+/CD133+/VEGFR-2+ cells are positive for the common leukocyte marker CD45 and therefore are likely to represent hematopoietic rather than endothelial precursors [18]. On the other hand, FACS is a relatively sensitive technique but with rapid advancement in technology; instead of MACS, FACS has not only gained significant attention but is now the mainstay to isolate, classify, and analyse progenitor cells because of its versatility and ease in obtaining a high percentage of pure populations [19,20].

In our research group, EPCs have been identified for the presence of specific surface antigens (CD34, VEGFR-2 and CD133). The samples were acquired by FACS with the protocol of the International Society of Hematotherapy and Graft Engineering (ISHAGE) [20]. Moreover, early EPCs in vitro have been identified for their capacity to uptake acetylated low-density lipoproteins (acLDL) and to bind Ulex europaeus agglutinin-1 (UEA-1).

### 3.3. Tissue Characterization

After their mobilization from the bone marrow into the bloodstream, EPCs home to the myocardium where they seem to be associated with the reparative mechanisms taking place in the areas of ischemic injury [21,22]. In particular, we believe that EPCs accumulate and are stored in the atrial appendage of the myocardium, a well-known source of several precursor cells [23]. Here, EPCs are primarily located inside the endothelium or the interstitium. EPCs have been frequently defined as CD34 and VEGFR2 positive cells [24]. CD34+/VEGFR+ cells are believed to represent EPCs generated at sites of vascular injury from circulating CD34+ cells [22,23]. EPCs are easily distinguished from mature endothelial cells which do not express VEGFR-2. The identification of EPCs is performed by immunohistochemistry: 3-μm sections from properly processed atrial appendage fragments are incubated with monoclonal antibodies (anti-CD34, anti-VEGFR-2) together with opportune enzymes and chromogen substrates (AEC, red for CD34; BCIP/NBT, blue for VEGFR-2). Together with morphology, the co-expression of these two markers allows EPCs to be identified and counted. EPCs are quantified by light microscopy, counting the number of double positive/purple, medium sized/spindle cells/mm^2^ present in the myocardial tissue from the atrial appendage [23].

### 3.4. Isolation in the Operatory Room

In our research group, we decided to study EPCs by isolating them from fragments of the right atrial appendage obtained during the institution of cardiopulmonary bypass (CPB). For this purpose, all patients scheduled for open heart procedures, performed either through a complete or mini-sternotomy approach, could be enrolled. After the opening of the sternum and pericardium, systemic heparinization was administered. After an activated clotting time of >480 sec was reached, cannulation for CPB was carried out using the ascending aorta or proximal arch for systemic perfusion and the right atrium or both venae cava for venous drainage. Independent of the type of venous cannulation, a sufficiently large purse string suture is placed at the base of the right auricle. Prior to insertion of the venous cannula, the tip of the right auricle is cut obtaining an adequate sample of atrial tissue which is stored in a small sterile container filled with formaldehyde solution and immediately sent for analysis. The cannula is then inserted into the resulting right atrial opening and secured by snaring the purse string, providing local hemostasis at the same time. This technique is simple, fast and low risk, while, when done accurately, it neither alters the routine institution of CPB, increases the operative time, nor is associated with dismal complications which could adversely affect patient outcome.

## 4. EPC from Bench to Bedside Literature Review

### 4.1. The Pathophysiologic Role in Aging

**Figure 1:**
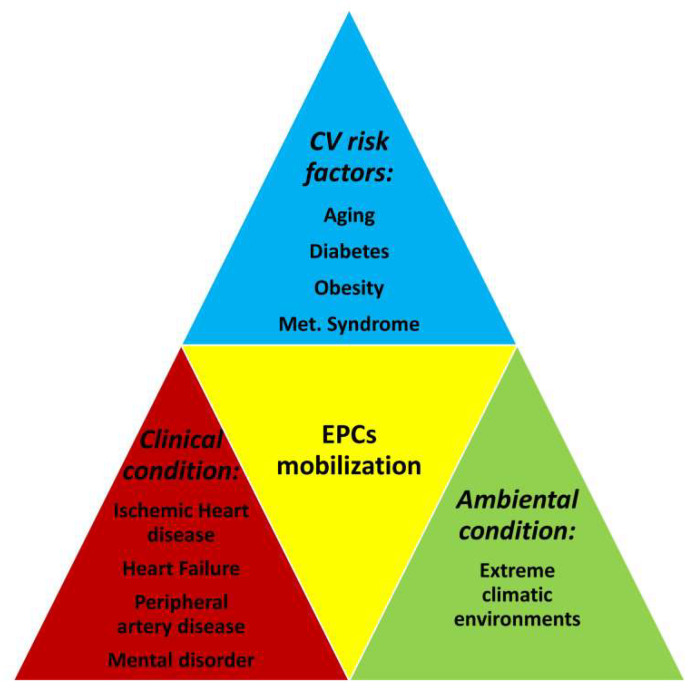
A snapshot describing the various clinical conditions associated with EPC mobilization.

Aging is a physiological multifactorial process that induces a gradual decline of the functions of organs, tissues and cells [25]. In particular, senescence of ECs leads to endothelial dysfunction that is associates with common CVD [25,26]. For instance, it is known that in animal models of hind limb ischemia, blood flow recovery is impaired in aged animals [27] and that ECs from old mice have a reduced capacity to proliferate and migrate than those from young mice [28]. In addition, in a mouse model of ischemia, aging leads to an insufficient collateral circulation in many tissues, thus determining a more severe injury to ischemic tissues [29]. Moreover, older patients with coronary heart disease (CAD) show a reduced capacity for developing collateral arteries [30]. In this respect, the evidence that, after engraftment, EPCs may substitute old ECs with new ones indicates that EPCs may be considered an effective tool to potentially block or delay vessel damage induced by aging [31]. In this respect, it has been observed that drug treatments designed to treat CVD have favourable effects on the number of circulating EPCs and their function [32]. On the other hand, the number and function of circulating EPCs is inversely correlated with age, suggesting that senescence may impair their function. For instance, survival, proliferation and migration are reduced in EPCs from aged persons [33,34,35]. In addition, in a rat model of heart injury, the endothelial repair is impaired more in older than in younger animals [36]. The molecular mechanisms linking aging with the reduction of number and function of circulating EPCs are not completely known, although they are likely to involve an altered balance between factors promoting cell growth, proliferation and migration, and factors promoting oxidative stress as well as senescence and/or apoptosis [37]. Interestingly, there is evidence that, both in humans and in mice, EPC dysfunction can be reversed by growth hormone (GH)-mediated increase of insulin-like growth factor 1 (IGF-1) levels [34]. In addition, it has been demonstrated that culturing EPCs from old rats in serum from young rats promotes the recovery of EPC activity, likely through the involvement of the phosphatidylinositol-3-kinase/Akt pathway [37]. Moreover, the activation of the nuclear factor (erythroid-derived 2)-like 2 protects EPCs from aged mice against oxidative stress, thus ameliorating their biological dysfunction [38]. Taken together, these findings are the proof of concept that EPC senescence may be counteracted by appropriate treatments aimed at slowing down the decline in EPC function during aging and that treatments stimulating EPCs in elderly people may have clinical implications for the aging population, Figure 1.

### 4.2. Diabetes and Obesity: The Case of Metabolic Syndrome

The natural process of aging can be accelerated by stress conditions, such as those characterizing metabolic diseases, type-2 diabetes and obesity [39]. In this respect, in vitro studies have demonstrated that hyperglycaemia accelerates the senescence of EPCs, decreasing their migration in response to homing factors, their vessel-forming capability and their nitric oxide synthase activity [40,41,42]. In addition, in vivo studies indicate that EPC mobilization is impaired in diabetic mice [43] and that EPC number and function is reduced in patients suffering from type-2 diabetes [44]. Moreover, there is evidence from studies performed in animal models as well as in humans that obesity reduces EPC proliferation rate and mobility, and suppresses the capability of EPCs to home to sites of vessel damage and to promote vessel repair [43,45]. Our group has shown that the presence of moderate pre-diabetic hyperglycaemic states is characterized by a reduction of the number of circulating EPCs and by an inverse correlation between the level of EPCs and fasting as well as post-OGTT plasma glucose concentrations [46]. In another study in vitro, we found that EPCs have a well-adaptive response to oxidative stress induced by constant and sustained high glucose exposure, conditions in which they seem to be more resistant than mature ECs [47]; this resistance to high glucose levels might be due to increased expression and activity of glutathione peroxidase allowing better cell survival [47]. In young subjects with relatively long-lasting type 1 diabetes mellitus, our group found a generalized preclinical involvement of large artery structure and function as well as a blunted endothelium regenerating capacity. Hyperglycemia and suboptimal chronic glycemic control seem to deteriorate functional arterial characteristics, such as large arteries stiffness, wave reflection and peripheral endothelium-dependent vasodilation, whereas an impaired endothelium regenerating capacity and adiponectin levels seem to influence arterial structure [48].

In recent decades, metabolic syndrome (MS) has become a very common syndrome globally. It refers to a cluster of morbidities including central obesity, high blood pressure, dyslipidaemia and impaired glucose metabolism with insulin resistance and adiposity as central features, which increases the risk for CVD and type 2 diabetes [49]. It has been observed that the number and function of circulating EPCs are reduced in patients affected by MS [50,51,52,53,54]. The pathogenic mechanisms through which MS (as well as obesity and/or type 2 diabetes) affects EPCs are not clearly delineated so far. The reduction in EPC number and function may be secondary to oxidative stress, nitric oxide availability and alterations in functioning of intracellular signaling pathways. In addition, an increased turnover of EPCs that may lead to replicative senescence cannot be excluded. Results from animal models of MS also point to a possible role of apoptosis in generating a decrease in EPC levels, as well as in EPC proliferation potential [55]. It is noteworthy that drug treatments counteracting the morbidities characterizing MS, such as anti-hypertensive or anti-diabetic drugs, as well as lifestyle approaches, such as moderate exercise or healthy nutrition, may induce ameliorative effects on EPC biology, suggesting that prophylactic measures influencing EPC proliferation, migration and homing may be viewed as possible treatment options in patients suffering from MS.

### 4.3. EPC Role in Ischemic Myocardium: ACS and SCAD

The pathophysiology process of ischemic heart disease is a complex interplay of different factors. EPCs are directly correlated with endothelial function and inversely correlated with cardiovascular risk factors and atherosclerosis progression [9,56]. EPC levels have also been correlated to prognosis evaluation in cardiovascular disease [57]. The involvement of EPC in acute coronary syndrome (ACS) has been largely discussed. The first correlation was reported by Shintani et al.; in patients with myocardial infarction (MI), they identified a peak in EPC levels after seven days with a statistically significant difference as compared to controls [56]. In another study, EPC levels were significantly higher in MI patients at admission than in controls [58], with EPC numbers significantly lower on day seven than at admission but still higher than in control patients. Although the involvement of EPCs in ACS has been largely discussed [58] and demonstration of EPC mobilization into circulation has been supported by strong scientific evidence in patients with acute MI and ACS [59], little evidence is available regarding the context of stable ischemic heart disease (SCAD). Recent advances in molecular EPC mechanisms highlight the involvement of different growth factors and signaling pathways in EPC mobilization and differentiation into mature ECs: in detail, VEGF and SDF-1 seem to play an important role as chemoattractants in the mobilization and homing of progenitor cells, also serving as EC differentiation factors; moreover, CD34+ cells can rapidly translocate KDR, whose expression is crucial for vascular development, from intracellular storage organelles to the cell surface in a regulated phosphatidylinositol-3-kinase (PI3K)-dependent pathway [24,60]. The presence of CAD and the consequent chronic ischemia could represent a trigger to increase EPCs recruitment through mobilization from bone marrow and homing in the myocardium, supporting the hypothesis of EPC involvement in the reparative mechanisms of the ischemic myocardium [24,61]. Morrone et al. showed that the number of EPC in the tissue of patients with CAD is significantly higher when compared with control subjects (*p* < 0.005), and circulating EPCs show a tendency to be reduced by approximately 20% in peripheral blood of patients with CAD when compared with non-CAD patients. The concentration of EPCs is higher in patients with coronary disease when corrected for risk factors (*p* = 0.035) [23]. In line with these results, this study is an integration to previously published articles and a little step forward in elucidating current controversies in this field. 

### 4.4. Heart Failure

Heart failure (HF) is a prevalent disease with high mortality and morbidity rates [62] characterized by loss of cardiomyocytes leading to cardiac fibrosis and dysfunctional cardiac remodelling that culminates in organ failure [63]. Endothelial dysfunction plays a crucial role in its pathogenesis, with its degree independently associated with poor clinical outcomes such as acute decompensation, repeat hospitalization, cardiac death, and transplantation [64]. 

In HF, endothelial dysfunction is a result of the imbalance between NO production and oxidative stress; this leads to reduced circulating NO and a lower level of circulating EPCs. This is supported by multiple studies [64,65,66] and is associated with circulating EPCs becoming functionally impaired [66]. Studies have highlighted a reduction in circulating EPCs associated with HF [63,64,65]. Circulating EPC levels also display an inverse correlation with NYHA functional class [65,67]. Functional ischemia induced by systolic dysfunction in HF and a chronic inflammatory state both impose myelosuppressive effects, which lead to a reduction in the mobilization of EPCs into the circulation. Furthermore, there is a reduction in the half-life of EPCs, which further drives dysfunctional ventricular remodelling and adverse outcomes [65]. Michelucci et al. reported an inverse relationship between circulating EPCs and the degree of abnormal cardiac remodelling evaluated by echocardiographic parameters [67]. As EPCs possess vasculogenic properties and play an important role in the maintenance of endothelial integrity, reductions in circulating EPCs may be associated with a suboptimal reparative response to endothelial damage. One experimental study demonstrated that neoangiogenesis by EPCs inhibits the apoptosis of hypertrophied myocardium, minimizes abnormal collagen deposition and scar formation, and optimizes ventricular function [68]. However, the exact mechanisms that underlie poor bone marrow mobilization and the reduction of EPC half-lives have yet to be fully elucidated. Kissel et al. [69] demonstrated impairments of EPC function in ischemic HF; whether reduced circulating EPCs and the suppressed marrow response are due to the underlying atherosclerotic process resulting in end-organ ischemia or as a direct consequence of HF itself has yet to be answered. Valgimigli et al. [66] demonstrated a biphasic response of EPCs with an increased production in the early stages of HF (NYHA I–II) due to the upregulation of the bone marrow response to endothelial dysfunction, followed by a reduction in EPC counts in late HF (NYHA III–IV). Therefore, EPC levels could potentially be used as a prognostic biomarker in patients with HF.

### 4.5. Peripheral Artery Disease

Peripheral artery disease (PAD) is commonly referred to as the ischemia of limbs associated with atherosclerotic lesion; its clinical manifestations (such as intermittent clau-dication) reflect the consequences of a mismatch between blood supply and demand. Critical limb ischemia (CLI) is the most severe clinical manifestation of PAD and, if not interrupted, could lead to ischemic ulcerations or even gangrene [70]. Traditional treatment strategies of CLI are focused on surgical bypass or endovascular interventions aimed at restoring perfusion and preventing amputation of the affected limb. However, a significant percentage of CLI patients do not have revascularization options, have poor prognosis and often require amputation. The therapeutic efficacy of EPCs has been not only documented in studies of CVD but also in PAD. In preclinical studies, the most adopted animal model is the hind limb ischemia model (HLI) [71]. In the HLI model, the femoral artery is ligated to reduce the blood supply to the lower leg, which induces angiogenesis to compensate for the reduced blood flow [72]. Lara-Hernandez et al. [73] showed that intramuscular administration of EPCs into the ischemic limbs of 28 patients with no-option critical limb ischemia was safe and feasible. They used 50 mL of G-CSF mobilized blood and then selected for CD34+ and CD133+ cells. This treatment resulted in a significant reduction in the pain score of no option CLI patients with increased tissue perfusion and promoted angiogenesis; furthermore, no adverse effects were noted after a follow-up of 14 months [73]. We also investigated the effects of iloprost, a prostacyclin analogue, on EPC levels in vivo in CLI patients. Patients with stage III and IV CLI were treated with iloprost for four weeks, highlighting an improvement of clinical and instrumental parameters and a significant increase in EPC number in the whole population, irrespective of age, sex, disease stage or atherosclerosis risk factors [74]. Iloprost increases EPC number in peripheral blood in vivo and such an effect may have therapeutic relevance.

The relationship between EPC mobilization and inflammatory response induced by peripheral transluminal angioplasty (PTA) was evaluated in male patients with PAD and intermittent claudication [75]. CFU-EC, CRP, IL-6 and erythropoietin (Epo) significantly increased at day 1 and were significantly correlated. No increase in VEGF, TNF-alpha or GM-CSF was observed at this early evaluation. After three months, all values returned to baseline. This study demonstrates that a transient CFU-EC mobilization occurs after angioplasty closely related to the inflammatory reaction that follows vascular injury, suggesting that CFU-EC are part of this inflammatory response. Epo increase had been previously shown to be associated with CFU-EC mobilization [76,77]. The lack of correlation between CFU-EC and VEGF may suggest that ischemia could play a minor role in early CFU-EC mobilization in this setting.

### 4.6. Carotid Angioplasty: Our Experience

Carotid atherosclerotic plaque is the primary cause of ischemic strokes [78]. Arterial intima media thickness (IMT) has been proposed as a surrogate endpoint for the degree of atherosclerosis and its progression. Existing evidence substantiates that common ca-rotid artery IMT measurement correlates with the prevalence of atherosclerosis in other arteries. The relationship between the increase in IMT and the progression of CVD has also been investigated in large studies [79,80]. However, the underlying mechanisms of plaque instability in the carotid share remarkable similarities with the coronary vasculature [81,82]. EPC number [83] and EPC migration [84] have been identified as independent predictors of carotid IMT in asymptomatic subjects, so EPCs can be considered an independent predictor of early preclinical atherosclerosis. Few data are available on the effect of internal carotid angioplasty (IC-PTA) on circulating CD34+ and EPC (defined as CD34+ KDR+ cells) mobilization. We studied the effect of elective PTA in different vascular districts (IC-PTA and peripheral PTA, PPTA). Data showed a transient CD34+ and EPC mobilization together with an increase of VEGF and CRP levels following vascular injury, suggesting that it can be part of a PTA-induced inflammatory response.

### 4.7. Mental Disorder

An established risk factor recognized today in the pathogenesis of arteriosclerosis and CVDs is depression [85]. Classical literature on depression and CVD shows that depression confers a relative risk between 1.5 and 2.0 for the onset of coronary artery disease in physically healthy individuals and a relative risk between 1.5 and 2.5 for cardiac morbidity and mortality in patients with existing coronary artery disease [85]. Within this context, the reasons for which EPCs have been studied over the last decade stem principally from two major points: (1) low-grade inflammation is implicated in pathogenesis of depression and is associated with increased cardiovascular and all-cause mortality [86]; (2) depression is characterized by increased cardiovascular morbidity and mortality that cannot be explained by traditional cardiovascular risk factors [87,88].

For these reasons, depressive disorders have been hypothesized to be associated with dysfunction of the immune system and the bone marrow. In particular, studies have been conducted to explore whether depression might influence the number of bone marrow-derived EPCs. Different research data on EPCs and depression emerged over the last decade and provided relatively consistent results. Dome et al. [89] demonstrated, for the first time, the evidence of a decreased number of circulating EPCs in a sample of patients with a current episode of major depression, suggesting that the connection between depression as a risk factor for cardiovascular disorders is mediated by its influence on the number of EPCs. Mature (CD34+) and immature (CD133+) EPCs counts were decreased in patients as compared with healthy subjects, and there was a significant inverse relationship between EPC levels and the severity of depressive symptoms. Consequently, impairment of the body’s EPC pool has been considered to have negative effects on the cardiovascular system, and patients with reduced numbers of EPCs are considered to be at increased risk for endothelial injury and for arteriosclerotic plaque development.

Chen et al. [90] explored the relationships between mature and immature circulating EPCs, expressing, respectively, CD34 and CD133 markers on the surface of cells and endothelial function in a sample of normal individuals without a significant risk of cardiovascular disease or major depression. The results indicated that even in the absence of clinical depression, mild levels of depression symptoms were associated with the depletion of circulating EPCs and endothelial dysfunction. In addition, the presence of a high depression score was associated with a depletion of circulating EPCs, but only of circulating mature but not immature EPCs. 

In a collaborative study carried out at the University of Pisa [91], the impact of depression on EPC levels in a sample of patients with a recent diagnosis of ACS was evaluated. About 50% of the patient sample was found to have suffered from a major depressive episode (MDE) across their lifespan. The authors also included in the study a sample of healthy subjects and a group of patients with depression without a history of cardiovascular disease. The results of the study showed that ACS patients with MDE had a significant decrease in circulating EPCs (i.e., CD133+, CD34+, KDR+, vasoregenerative, more immature EPC phenotype cells) as compared with ACS patients without MDE. In other terms, the presence of MDE may reduce the response of bone marrow to acute ischemic events and, therefore, cause decreased protection considering the reparative role of EPCs in ACS patients. 

Subsequently, in line with the results of Dome et al. [89] and Chen et al. [90], Felice et al. [92] investigated the relationship between the level of circulating EPCs (CD133+ CD34+ KDR+) and depression and anxiety in a much larger sample of patients who had recent acute coronary syndrome. The results of the study demonstrated that the group with mood or anxiety disorders showed a significant decrease in circulating EPCs when compared with the group of patients without affective disorders, corroborating their previous preliminary study [92]. Moreover, a negative correlation has emerged between EPC levels and severity of depression and anxiety, suggesting that the relationship between EPCs and psychopathology is not state-dependent but rather connected to the fact of suffering or not from a psychiatric disorder.

Lopez-Vilchez et al. [93] showed a significant elevation of different plasma markers of endothelial activation and damage in patients with major depression in comparison with healthy subjects. At the moment of diagnosis, they found increased levels of soluble VCAM-1, VWF and circulating CEC, and decreased numbers of circulating EPCs. These results were aligned with the existing literature. In this study, patients received treatment with the SSRI escitalopram over 24 weeks with the aim to explore the potential modulating effect of the antidepressant treatment. The authors noticed that there was a significant reduction in soluble VCAM-1 and VWF levels and in CEC counts in treated patients, showing a protective role in the modulation of the endothelial damage developed in these patients. There was also a tendency toward an increase in the number of EPCs, considered as an indicator of vascular repair, in the group of treated patients. 

Blum et al. [86] confirmed the presence of a decreased number of circulating EPCs along with high levels of VCAM-1 and VEGF (representing vascular inflammation and activation) in a sample of women with major depression without any depressive treatment as compared to healthy controls. The authors attributed responsibility to not just a decrease in the number but to a functional impairment in the ability to regenerate damaged blood vessels or create new blood vessels due to an impaired ability to build colony-forming units of endothelial progenitor cells (CFU-EPCs). This lack of regenerative capacity, impairment of the bone marrow to produce and mobilize EPCs from the bone marrow and to transfer them to the areas of need might cause the higher rate of cardiovascular complications observed in patients with depression.

According to the data summarized above, there are promising and detailed results regarding the relationships between cardiovascular disorders, affective disorders and EPCs (essentially, depression, which is the most frequent psychiatric disorder in the general population). It is plausible that reduced levels of circulating EPCs contribute substantially to the mediation of this triangular relationship. Future prospective studies are recommended in this area in patients with depressive disorders and in individuals with anxiety disorders or other psychiatric conditions [94].

The presence of affective disorders (which, by definition, are persisting or recurrent conditions in the majority of cases) may have a pathoplastic effect on the bone marrow response to acute cardiac ischemic events. This issue has been crucially investigated by the Pisa group within the framework of the collaborative study on EPCs, depressive disorders and ischemic heart disease [91,92,95]. Further research is strongly warranted in this area. Mechanisms underpinning bone marrow’s response to myocardial infarction in de-pressed patients may be either biologically connected to the psychiatric condition or to the multiple behavioral consequences of depression. For example, it is well known that altered psychomotricity and sedentary lifestyle are associated with depression, and exercise has been shown to stimulate the mobilization of EPCs [96]. It has also been observed that acute respiratory distress is associated with altered levels of circulating EPCs [97]. This association may be negatively affected in certain critical clinical situations by the presence of acute panic-agoraphobic symptoms with respiratory expressivity (dyspnea, choking feeling, suffocation sensations).

Considering the reparative role of EPCs in cardiac ischemic tissue, cardiological patients who are clinically depressed or anxious may be less protected than patients without mood or anxiety disorders from the risk of negative course after an acute coronary syndrome and may even be prone to the occurrence of new major cardiac events. 

### 4.8. Exposure to Extreme Climatic Environments

An interesting setting is the behavior of EPCs in extreme climatic exposure. Our group evaluated the response of circulating EPCs to extreme temperature in volunteers participating in a two-month long Antarctic mission, according to the Italian Antarctic Research Programme (PNRA, Programma Nazionale di Ricerche in Antartide). Circulating EPCs (defined as triple positive CD133/CD34/VEGFR2 cells) were evaluated by flow cytometry analysis before and after the mission. In addition, plasma levels of stem cell factor/c-kit ligand (SCF), a strong chemo-attractive factor for EPCs, was evaluated. All volunteers were free of CV risk factors and none of them showed differences in clinical or cardiovascular assessment after the mission. However, circulating EPC numbers were significantly reduced after the mission. On the contrary, plasma levels of SCF were significantly increased after the mission. Based on these results, we hypothesize that the reduction observed in EPC levels after the Antarctic mission can be due to the fact that extreme low temperature increased plasma levels of SCF which can exert their chemo-attractive function on EPCs at the damaged vascular site, not clinically detectable, thus reducing their circulating levels. These results deserve attention, since even though these subjects do not have any clinical evidence of cardiovascular damage, the presence of reduced levels of EPCs could be an early indicator of cardiovascular subclinical damage that should be monitored strictly [98].

## 5. Conclusions

As reviewed here, EPCs appear to play an important role in revealing functional alterations induced by cardiovascular diseases. In this respect, there are findings demonstrating that EPCs are able to detect early cardiovascular alterations not yet diagnosed through currently used techniques. In fact, low blood levels of EPCs are indeed early signs of endothelial dysfunction. Most of the findings reviewed here concur that reduced blood levels of EPCs would be an early sign of cardiovascular compromise. To summarize, in Table 1 we showed the measurement of EPCs (whether they were found to be increased or decreased), the markers used to identify them and the different approaches used reported in the present review. 

To summarize the EPC physiopathology, EPCs are produced by bone marrow to be then released in the bloodstream, from where, through the “homing” process, they migrate to tissues. The first question to be answered is whether reduced blood levels of EPCs in cardiovascular diseases might reflect their decreased production by bone marrow or their increased “homing” to tissues. In cardiovascular patients, simultaneous measurements of blood and tissue levels of EPCs revealed that reduced blood values of EPCs was dependent on an increased “homing” process. The second question to be answered is related to the “homing” mechanism and to its physiopathological significance. By migrating to damaged tissues, EPCs should play a reparatory role by promoting neoangiogenesis in ischemic tissues. This hypothesis has been successfully proven by preliminary data in ischemic heart diseases, although much work is needed to confirm this possibility. In this respect, potentiating EPC function might serve as a novel therapeutic approach against ischemic diseases. Although extensively studied, EPCs still have dark sides that need to be clarified by further work and in-depth studies.

## Figures and Tables

**Table 1:** EPC measurement and variation.

Study	Clinical Setting	Study Size (n. of Patients)	EPC Determination	Phenotype	Main Results (Number and Functionality of EPCs)
Williamson et al. [32]	Aging	-	Isolation from cord and peripheral blood of young (20–30 years) and old (60–70 year) subjects and expanded in vitro	Outgrowth endothelial cells (OECs): cobblestone morphology, VE-CAD+/vWF+/CD31+/CD34+/CD146+, able to incorporate acetylated-LDL, to bind the lectin UEA-1 and to form a network of tubelike structures	Proliferative, survival and tube-forming capacity of OECs is not significantly impaired with age. Migratory response of OECs declines with age (migratory capacity of human OECs toward VEGF and SDF-1a significantly declines with increasing age. Furthermore, the reduction in the chemotactic response of OECs significantly correlates with structural alterations of HS).
Thum et al. [33]	//	-	Circulating EPC number and function of middle-aged subjects before and after treatment with recombinant growth hormone (IGF-1) compared with young controls	CD34+/CD133+/CD117+ and CD133+/VEGFR2+ peripheral cells	Middle-aged and elderly subjects had lower circulating CD133+/VEGFR-2+ EPCs with impaired function and increased senescence. GH treatment in middle-aged subjects elevated IGF-1 levels, increased circulating EPCs with improved colony forming and migratory capacity, enhanced incorporation into tube-like structures, and augmented endothelial nitric oxide synthase expression in EPCs comparable to that of the younger group.
Heiss et al. [34]	//	40	Circulating EPC number and function in 20 young (25 ± 1 year) and 20 old (61 ± 2 years) healthy subjects without clinical evidence of other CV risk factors	KDR+/CD34+/or KDR+/CD133+ circulating cells, KDR+/CD31+/CD105+/CD146+/vWF+ cells culture	There were no differences in the numbers of circulating EPCs. Older subjects had significantly impaired endothelium-dependent dilation of the brachial artery (flow-mediated dilation, FMD) in addition to a lower survival, migration, and proliferation capacity.
Chen et al. [41]	Diabetes	-	Early and late EPCs by MNCs isolated from healthy subjects incubated with glucose/mannitol or drugs	Early EPCs: adherent cells double positive for acetylated LDL (acLDL) uptake and lectin binding Late EPCs: VE-CAD+/vWF+/PECAM-1+/CD31+/CD34+/KDR+)/VEGFR2+/CD133+	High glucose reduced the number and proliferation of early and late EPCs, enhanced EPC senescence and impaired the migration and tube formation of late EPCs. The effects of high glucose could be ameliorated by coincubation with an NO donor.
Fadini et al. [46]	//	219	Circulating EPCs and progenitor cells (PCs) isolated from the peripheral blood of individuals with carbohydrate metabolism abnormalities	CD34+/KDR+ (EPCs) and CD34+ (PCs)	Circulating PCs and EPSs were inversely related to glucose tolerance.
Felice et al. [47]	//	-	Isolated from total peripheral blood of healthy male donors (age 40 years) after 3 days of culture in a selective medium	Adherent cells positive for DiI-ac-LDL/Lectin and KDR+/VE-CAD+/vWF+/CD31+/C14+	EPCs have a well-adaptive response to oxidative stress induced by constant and sustained high glucose exposure, maybe due to increased expression and activity of glutathione peroxidase allowing better cell survival.
Palombo et al. [48]	//	42	Circulating EPCs and progenitor cells (PCs) isolated from peripheral blood of 16 uncomplicated young T1DM pts (mean age 18 ± 2 years) and 26 controls	CD34+/KDR+ (EPCs) and CD34+ (PCs)	Young subjects with relatively long-lasting T1DM free of overt clinical complications have a significantly lower count of circulating EPCs and a generalized preclinical involvement of large artery structure and function as compared to healthy controls.
Berezin et al. [51]	Met. syndrome	47 + 35	Evaluation of circulating EPCs/MPCs in 47 patients with MetS without known CV disease and 35 healthy volunteers	CD45-/CD34+ (EPCs) and CD14+/Tie-2+ (MPCs)	Depletion of numerous circulating EPCs and MPCs in MetS patients was found.
Jialal et al. [53]	//	31 + 46	Enumerate and functionally characterize EPCs in subjects with Met S. in comparison to healthy controls	KDR+/CD34+, functionality was assessed by CFU assay, migration and tubule formation.	MetS subjects without diabetes or CVD have decreased EPC numbers and impaired functionality as compared to control subjects.
Westerweel et al. [54]	//	29	Evaluation of circulating EPCs in subjects with obesity and MetS; evaluation of the effect on EPC levels of two lipid-lowering treatments	CD34+/KDR+	EPC levels are reduced in apparently healthy men with abdominal obesity and metabolic syndrome. Intensive lipid-lowering treatment increased circulating EPCs to control levels.
Shintani et al. [56]	ACS	16 + 8	Evaluation of EPCs and MNCs in in patients with acute myocardial infarction (16) and control subjects (8)	EPCs: CD34+/KDR+/VE-CAD+/CD31+/DiI-acLDL+/CD45- MNCs: CD34+	This is the first clinical demonstration showing that lineage-committed EPCs and MNC (CD34+) cells, their putative precursors, are mobilized during an acute ischemic event in humans.
Massa et al. [57]	ACS and SCAD	26 + 10 + 17	Phenotypic and functional analysis of circulating CD34+ HPCs in patients with acute myocardial infarction (AMI) assessed from admission up to 60 days, in patients with stable angina pectoris (SA), and in healthy controls	HPCs: CD34+/CD45- EPCs: CD34+/VEGRF-2+/CD31+/VE-CAD+	Spontaneous mobilization of both HPCs and EPCs occurs within a few hours from the onset of AMI and is detectable until 2 months.
Morrone et al. [23]	SCAD	55	Investigate the association of stable ischemic heart disease with EPC levels in tissue and blood	CD34+/KDR+	Patients with stable CAD had higher EPC density values (EPC/mm^2^) and were more likely to have lower EPC blood levels when compared with normal controls.
Samman et al. [64]	Heart failure	514	Evaluation of the number of circulating PCs and the etiology and severity of HF and HF events	PCs: CD45med+/CD34+/CD133+/VEGF+/CXCR4+ (Endothelial-enriched PCs: CD34+/VEGF+)	PC levels are lower in patients with HF, and lower PC counts are strongly and independently predictive of mortality.
Sandri et al. [65]	//	120	Evaluation of number and function of EPCs in both physiologic aging and chronic heart failure (CHF), evaluation of beneficial effects of exercise training	CD34+/KDR+	EPC numbers and function were lower in older and in CHF patients. Four weeks of exercise training was effective in improving EPC number and EPC function in CHF patients.
Michelucci et al. [67]	//	85	Evaluate the association between circulating PCs and EPCs and left ventricular (LV) remodeling in CHF	CPCs: CD34+, CD133+ and CD34+/CD133+ EPCs: CD34+/KDR+, CD133+/KDR+ and CD34+/CD133+/KDR+	Data suggest a correlation between higher number of circulating EPCs and a less severe pathological state, with more preserved LV function and a lower degree of ventricular remodeling in CHF patients.
Di Stefano et al. [74]	PAD	23	Investigate the effects of iloprost on EPC levels in vivo in CLI (critical limb ischemia) patients	Early EPC: adherent cells with double positivity ac-LDL/UEA; also CD14+/CD45+	Iloprost increases EPC number in peripheral blood in vivo; such an effect may have therapeutic relevance.
Chironi et al. [83]	//	84	To assess whether circulating EPCs can be considered as a cardiovascular risk marker before event has occurred	CD34+/KDR+	EPC numbers decreased in the presence of carotid, aortic or femoral plaque as compared to the absence of plaque; in the presence of three sites affected, the reduction was greater.
Di Stefano et al. [91]	Mental disorder	107	Evaluate the impact of depression on EPC levels in ACS patients	CD34+/CD133+/KDR+	ACS patients with MDE (major depressive episode) have a reduced number of circulating EPCs compared with ACS patients without MDE.
Felice et al. [92]	//	111	Evaluate the impact of depression and anxiety on EPC levels in ACS patients	CD34+/CD133+/KDR+	The study indicates that EPCs circulate in decreased numbers in ACS patients with depression or anxiety.
Felice et al. [98]	Extreme climatic exposure	6	Evaluate the response of circulating EPCs, SCF, SDF-1, ET-1 and sICAM-1 to extreme temperature in volunteers participating in a 2 month Antarctic mission, according to the Italian Antarctic Research Programme (PNRA).	CD34+/CD133+/KDR+	Circulating EPC numbers were significantly reduced after the mission, while plasma levels of SCF were significantly increased after the mission.
